# Seventeen New Complete mtDNA Sequences Reveal Extensive Mitochondrial Genome Evolution within the Demospongiae

**DOI:** 10.1371/journal.pone.0002723

**Published:** 2008-07-16

**Authors:** Xiujuan Wang, Dennis V. Lavrov

**Affiliations:** Department of Ecology, Evolution and Organismal Biology, Ames, Iowa, United States of America; Max Planck Institute for Evolutionary Anthropology, Germany

## Abstract

Two major transitions in animal evolution–the origins of multicellularity and bilaterality–correlate with major changes in mitochondrial DNA (mtDNA) organization. Demosponges, the largest class in the phylum Porifera, underwent only the first of these transitions and their mitochondrial genomes display a peculiar combination of ancestral and animal-specific features. To get an insight into the evolution of mitochondrial genomes within the Demospongiae, we determined 17 new mtDNA sequences from this group and analyzing them with five previously published sequences. Our analysis revealed that all demosponge mtDNAs are 16- to 25-kbp circular molecules, containing 13–15 protein genes, 2 rRNA genes, and 2–27 tRNA genes. All but four pairs of sampled genomes had unique gene orders, with the number of shared gene boundaries ranging from 1 to 41. Although most demosponge species displayed low rates of mitochondrial sequence evolution, a significant acceleration in evolutionary rates occurred in the G1 group (orders Dendroceratida, Dictyoceratida, and Verticillitida). Large variation in mtDNA organization was also observed within the G0 group (order Homosclerophorida) including gene rearrangements, loss of tRNA genes, and the presence of two introns in *Plakortis angulospiculatus*. While introns are rare in modern-day demosponge mtDNA, we inferred that at least one intron was present in *cox1* of the common ancestor of all demosponges. Our study uncovered an extensive mitochondrial genomic diversity within the Demospongiae. Although all sampled mitochondrial genomes retained some ancestral features, including a minimally modified genetic code, conserved structures of tRNA genes, and presence of multiple non-coding regions, they vary considerably in their size, gene content, gene order, and the rates of sequence evolution. Some of the changes in demosponge mtDNA, such as the loss of tRNA genes and the appearance of hairpin-containing repetitive elements, occurred in parallel in several lineages and suggest general trends in demosponge mtDNA evolution.

## Introduction

Two major evolutionary events occurred early in animal history and shaped the majority of animals, as we know them today: the origin of multicellularity and the origin of bilateral symmetry. The phylogenetic boundaries of these events are well defined among extant taxa and correspond to the traditional groups Metazoa (multicellular animals) and Bilateria (all animal phyla except Porifera, Placozoa, Cnidaria, and Ctenophora). Multiple genomic changes must have occurred in association with these morphological transitions, and current genome sequencing projects give us the first glimpses into these changes [1], [2].

Surprisingly, the transitions to multicellular and bilaterally symmetrical animals also correlate with multiple changes in mitochondrial genome architecture [3], although the main function of mitochondria themselves remained unchanged. In particular, the origin of animal multicellularity is associated with the loss of all ribosomal protein genes from mtDNA, the disappearance of most introns, and a large reduction in the amount of non-coding DNA [3]. The origin of bilaterality correlates with further compaction of mtDNA, multiple changes in the genetic code and the associated losses of some tRNA genes, along with the appearance of several genetic novelties [4]. Obviously, the picture presented above is an extrapolation of our knowledge of extant organisms into the ancient past and as such can be affected by artifacts of ancestral state reconstruction [5]. It is also based on a relatively limited sampling of mitochondrial genomes, especially from non-bilaterian animals, and additional data from Cnidaria, Ctenophora, Porifera, as well as the closely related lineages of eukaryotes (e.g., Choanozoa) are essential to support, expand, or refute it.

**Figure 1:**
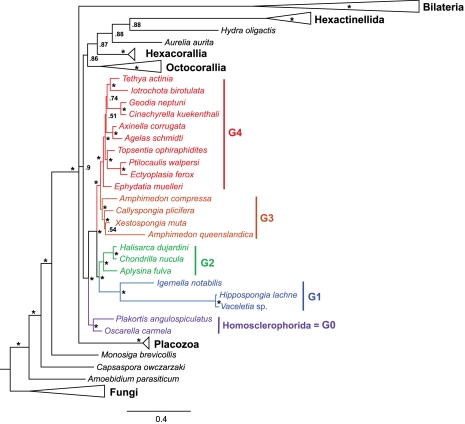
Phylogenetic analysis of demosponge relationships using mitochondrial genomic data. Posterior majority-rule consensus tree obtained from the analysis of 2,558 aligned amino acid positions under the CAT+F+Γ model is shown. Other methods of phylogenetic reconstruction produced similar topologies [11]. The numbers at each node are Bayesian posterior probabilities. Nodes with ≥95% support are marked with an asterisk. For simplicity, non-demosponge clades were collapsed to triangles. The full tree is presented in Figure S1.

Class Demospongiae [6] is the largest (>85% of species) and most morphologically diverse group in the phylum Porifera. It contains sponges of various shapes and sizes that occupy both freshwater and marine environments from shallow to abysmal depths and includes such oddities as carnivorous sponges [7]. Within the extant Demospongiae 14 orders are recognized that encompass 88 families, 500 genera and more than 8000 described species [8], [9]. Although traditionally three subclasses have been distinguished, two of them do not appear to be monophyletic. Instead, recent molecular studies [10], [11] provide strong support for five major clades within the Demospongiae: Homoscleromorpha (G0) (Homosclerophorida), Keratosa (G1) (Dictyoceratida+Dendroceratida), Myxospongiae (G2) (Chondrosida, Halisarcida, and Verongida), Marine Haplosclerida (G3), and all the remaining groups (G4) (Figure 1). Our knowledge of mtDNA diversity within the demosponges has been rudimentary, with only five sequences representing 3 of the 5 major groups available [12]–[15]. Previous studies revealed that demosponge mtDNA resembles that of most other animals in its compact organization, lack of introns, and well-conserved gene order, but at the same time contains extra genes, including *atp9, trnI(cau), trnR(ucu),* encodes bacterial-like ribosomal and transfer RNAs, and uses a minimally derived genetic code in protein synthesis [12]. Furthermore, additional unusual features found in the mitochondrial genomes of *Oscarella carmela* [14] and *Amphimedon queenslandica* [15] suggested that more mitochondrial genomic diversity might exist among the demosponges. Here we describe complete mitochondrial sequences from 17 species of demosponges and analyze them with five previously published mitochondrial genomes from this group that were available at the time this study was conducted. Taken together, our sampling covers all recognized order-level diversity within the Demospongiae and provides the first analysis of general evolutionary trends in mitochondrial genome organization for this group. Such a comprehensive approach to the analysis of demosponge mtDNA is needed because, at least in the fossil record, the evolution of demosponges closely mirrors the evolution of all bilaterian animals with the first demosponge fossils appearing in Precambrian deposits and a major radiation occurring in the Lower Cambrian [16], [17].

## Results

### Genome organization and nucleotide composition

**Figure 2:**
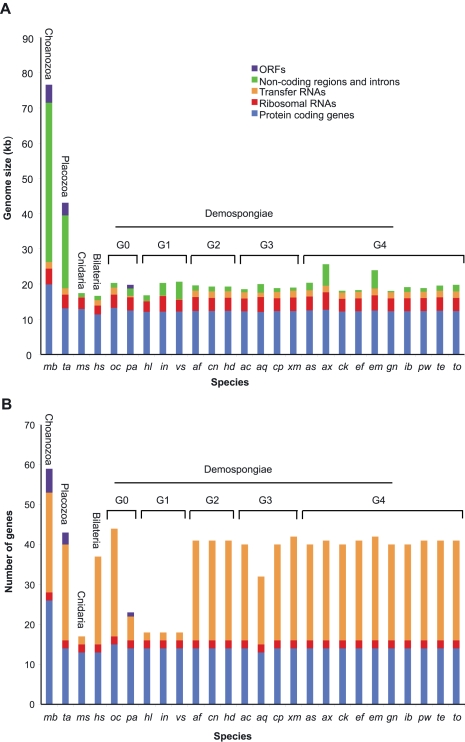
The size (A) and gene content (B) of demosponge mtDNA. Demosponge species are subdivided into five major groups (G0–G5). Selected species from other animal groups and the outgroup *Monosiga brevicollis* are included for comparison. Species are abbreviated as following: *mb*, *M. brevicollis*; *ta*, *Trichoplax adhaerens*; *ms*, *Metridium senile*; *hs*, *Homo sapiens*; *oc*, *Oscarella carmela*; *pa*, *Plakortis angulospiculatus*; *hl*, *Hippospongia lachne*; *in*, *Igernella notabilis*; *vs*, *Vaceletia* sp.; *af*, *Aplysina fistularis*; *cn*, *Chondrilla nucula*; *hd*, *Halisarca dujardini*; *ac*, *Amphimedon compressa*; *aq*, *Amphimedon queenslandica*; *cp*, *Callyspongia plicifera*; *xm*, *Xestospongia muta*; *as*, *Agelas schmidti*; *ck*, *Cinachyrella kuekenthali*; *ef*, *Ectyoplasia ferox*; *em*, *Ephydatia muelleri*; *gn*, *Geodia neptuni*; *to*, *Topsentia ophiraphidites*; *ib*, *Iotrochota birotulata*; *pw*, *Ptilocaulis walpersi*; *ax*, *Axinella corrugata*; *te*, *Tethya actinia*.

All twenty-two analyzed mtDNAs of demosponges were circular-mapping molecules, each containing a conserved set of thirteen protein-coding and two rRNA genes identical to that found in the mtDNA of most bilaterian animals [18]. In addition, *atp9*, a gene for subunit 9 of ATP synthase was identified in mtDNA of all demosponges except *Amphimedon queenslandica* [15], and *tatC,* a gene for twin arginine translocase subunit C, was found in *Oscarella carmela* [14]. The number of tRNA genes showed more variation. Although 24 or 25 tRNA genes were present in most analyzed demosponge mitochondrial genomes, as few as 2 and as many as 27 tRNA genes were found in mitochondrial genomes of some demosponge species (Figure 2, see below). In addition, a sequence with a potential to form a tRNA-like structure, named *trnX*, was located downstream of *cox1* in *Xestospongia muta* and *Ephydatia muelleri* mtDNA. Inferred tRNA(X) had a well-conserved primary (65.3% nucleotide identity) and secondary structure, except for the putative anticodon arm, which differed both in length and in sequence between the two species.

The sampled demosponge mitochondrial genomes displayed moderate size variation (16–26 kb; mean = 19.7 kb), most of which could be attributed to the expansions of non-coding regions usually caused by the presence of repetitive elements (Figure 2). We detected no obvious phylogenetic pattern associated with this variation, and no similarity in the sequence of repetitive elements among different species. Most demosponge mitochondrial genomes were larger than their counterparts in bilaterian animals. However, even the largest demosponge mitochondrial genomes were dwarfed in comparison to those in the choanoflagellate *Monosiga brevicollis* and the placozoan *Trichoplax adhaerens*, which have a much higher percentage of non-coding DNA and, in the case of *M. brevicollis*, an expanded gene set (Figure 2).

**Figure 3:**
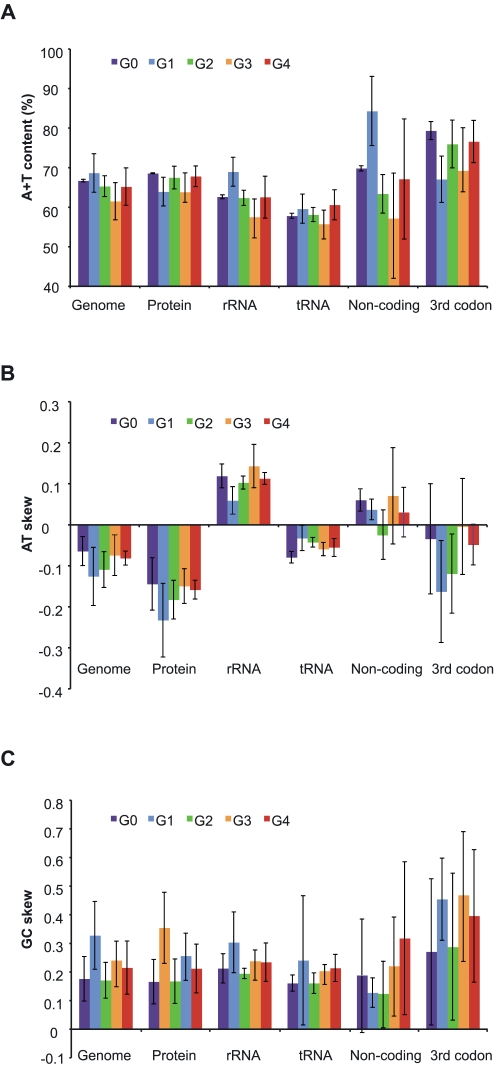
Nucleotide composition of mtDNA in five major groups of demosponges. (A) A+T content; (B) AT-skew; (C) GC-skew. The values are shown for the sense (non-template) strand of the whole genome (genome), its concatenated genetic components (protein genes, rRNA genes, and tRNA genes), 3^rd^ codon positions in protein genes, and for the corresponding strand in intergenic regions. Colored bars indicate the mean value for each group of demosponges; error bars show standard deviation.

All analyzed mitochondrial genomes were relatively uniform in the overall nucleotide composition (A+T content between 56–72%) and, on average, displayed negative AT- and positive GC-skews of the coding strand (Figure 3). The sense strand of protein and tRNA genes had a negative AT-skew in all species, that of rRNA genes had a positive AT-skew, while non-coding regions and 3^rd^ codon positions showed a large variation in AT-skew both among and within major demosponge groups (Figure 3B). All types of sequences in demosponge mtDNAs showed positive GC-skews except for the tRNA genes in *Igernella notabilis* and the non-coding regions in *Ephydatia muelleri* and *Aplysina fistularis*. The genomic values for AT- and GC-skews correlated more strongly with those for protein genes (R^2^ = 0.89 and 0.95, respectively) and rRNA genes (R^2^ = 0.61 and 0.93) than those for tRNA genes (R^2^ = 0.06 and 0.57) and non-coding regions (R^2^ = 0.13 and 0.34), while genomic A+T content correlated most strongly with that of rRNA genes (R^2^ = 0.89) comparing to non-coding regions (R^2^ = 0.78), tRNA genes (R^2^ = 0.65), and protein genes (R^2^ = 0.44). Interestingly, non-coding regions and 3^rd^ codons (that are usually assumed to experience similar mutational pressure) showed little correlation in all three types of measurements (R^2^ values are 0.05, 0.3 and 0.58 for A+T content, AT- and GC-skews, respectively).

**Figure 4:**
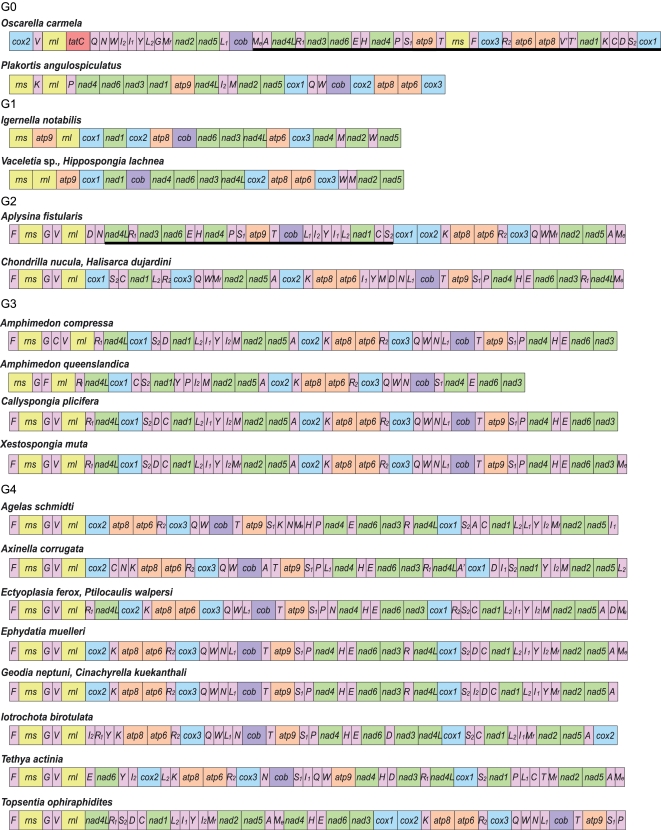
Mitochondrial gene arrangements in demosponges. Protein and rRNA genes (larger boxes) are: *atp6*, *8-9*–subunits 6, 8 and 9 of the F_0_ ATPase, *cox1-3*–cytochrome *c* oxidase subunits 1-3, *cob*–apocytochrome *b* (*cob*), *nad1-6* and *nad4L*–NADH dehydrogenase subunits 1-6 and 4L, *rns* and *rnl*–small and large subunit rRNAs, *tatC*–twin-arginine translocase component C. tRNA genes (smaller boxes) are abbreviated using the one-letter amino acid code. The two arginine, isoleucine, leucine, and serine tRNA genes are differentiated by subscripts with *trnR(ucg)* marked as *R_1_*, *trnR(ucu)*–as *R_2_*, *trnI(gau)*–as *I_1_*, *trnI(cau)*–as *I_2_*, *trnL(uag)*–as *L_1_*, *trnL(uaa)* as *L_2_*, *trnS(ucu)*–as *S_1_*, and *trnS(uga)*–as *S_2_*. All genes are transcribed from left to right except those underlined to indicate an opposite transcriptional orientation. Genes are not drawn to scale and intergenic regions are not shown.

All but four pairs of sampled mitochondrial genomes had unique gene orders, with the number of shared gene boundaries between individual genomes ranging from 1 to 41. The extent of gene order variation and the type of gene rearrangements differed among major groups of demosponges (Figure 4). Gene arrangements of protein and rRNA genes were generally well conserved within G2, G3 and G4 and the predominant type of change within these groups was tRNA transposition. By contrast, more rearrangements were found within G0 (13% of shared boundaries between two sampled genomes) as well as within G1 (59% of shared boundaries among three genomes). Still, most of the rearrangements were transpositions and only two inversions were found in the whole dataset (in *Oscarella carmela* and *Aplysina fistularis*).

### Protein coding genes

**Figure 5:**
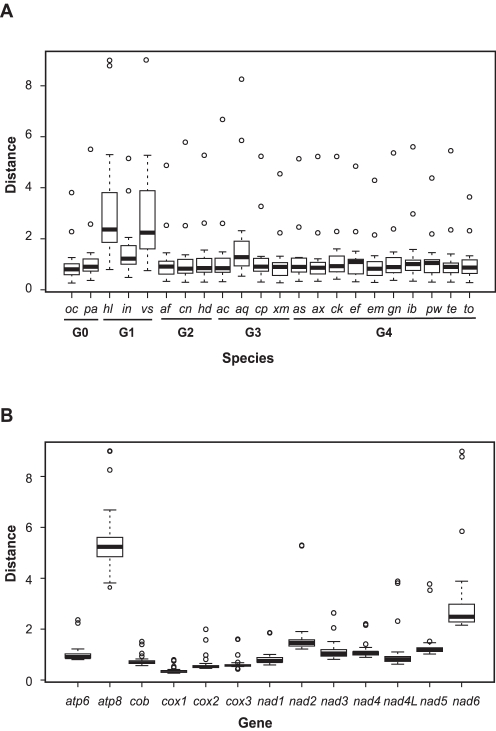
Relative rates of evolution of individual species (A) and individual genes (B). Rates are estimated by average genetic distances to orthologous genes from four cnidarians. Each boxplot represents data for 13 individual genes in (A) and 22 demosponge species in (B). Lower horizontal bar, non-outlier smallest observation; lower edge of rectangle, 25 percentile; central bar within rectangle, median; upper edge of rectangle, 75 percentile; upper horizontal bar, non-outlier largest observation; open circle, outlier.

The protein coding genes identified in all 22 demosponge mtDNAs showed 0.33–11.81% variation in size and 31.9–87.3% average pairwise identity calculated based on inferred amino acid sequences (Table S1). *Atp8* was the least conserved gene both in terms of size (11.81% variation), pairwise sequence identity among demosponges (31.9% on average, range 8.5–85.7%), and genetic distance to cnidarian homologues (Figure 5), followed by *nad6*. By contrast, *atp9*, a gene encoding another subunit of the ATP-synthase complex, was the most conserved, with an average pairwise identity of 87.3% (range 76.9%–100%). Other genes were relatively uniform both in their average pairwise identities across the demosponges and the calculated rates of sequence evolution (Figure 5).

Codon usage in all analyzed demosponge mitochondrial genomes was consistent with the minimally modified genetic code inferred in our previous study [12]. All 22 mtDNAs share similar codon usage bias with an effective number of codons equivalent to 41.8±3.5. Synonymous codons ending with A or T were clearly preferred (56–85% for individual species; 73.6% on average), while the codon CGC was not used at all in mitochondrial coding sequences of 12 species. *Tethya actinia* displayed the most biased mitochondrial codon usage with no AAC, CGC, CTC, CTG, and TGC codons present.

ATG was the most common initiation codon, followed by GTG, which occurred frequently in *nad6* (15 out of 22 species) and occasionally in other genes (Table S2). The unusual start codon ATT was inferred for *cox2* in *Hippospongia lachne*, *nad3* in *Cinachyrella kuekenthali* and *nad6* in *Vaceletia* sp. and a TGG start codon was inferred for *nad2* in *Ephydatia muelleri*, *nad6* in *Tethya actinia*, *Axinella corrugata*, *Amphimedon queenslandica* and *tatC* in *Oscarella carmela* (Table S2). Such initiation codons are common in mitochondrial coding sequences of bilaterian animals [4], but are rare, although not unprecedented, in non-bilaterian animals and non-animal outgroups [19], [20]. The stop codons TAA and TAG were inferred for all coding sequences except *nad5* in *Amphimedon compressa*, *Ectyoplasia ferox*, *Ephydatia muelleri*, and *Callyspongia plicifera* as well as *nad4L* in *Cinachyrella kuekenthali.* No standard or abbreviated stop codons were found for the latter genes and the mechanism of their translational termination remains unclear.

Among the five major clades within the Demospongiae (G0–G4), a significant acceleration in the rates of evolution was found in G1, especially in the lineage leading to *Vaceletia* sp. and *Hippospongia lachne* (Figure 5; RRTree P = 1.00E−07). We tested whether the G1 accelerated rates could have been the result of positive selection as suggested by Bazin *et al.* [21] but did not find significant support for this hypothesis by either the M1–M2 test in PAML or by the synonymous vs. non-synonymous substitution rate test with the DNASP program [22].

### Introns in cox1

Although introns are common in mtDNA of two groups of non-bilaterian animals, Cnidaria and Placozoa, only one mitochondrial intron (in *cox1* of *Tetilla* sp.) has been reported so far in demosponges [23]. Among the 22 demosponge mitochondrial genomes analyzed for this study, we found two additional group I introns, both of them in *cox1* of *Plakortis angulospiculatus*. These introns were 388 bp and 1118 bp in size (henceforth intron 1 and 2, respectively), and separated by only 9 nucleotides (3 codons) in the gene. Intron 2 in *P. angulospiculatus* was found after position 726 in *cox1*, at the same location as the intron reported for *Tetilla* sp. [23]. Intron 2 in *P. angulospiculatus* and its counterpart in *Tetilla* sp. share 81.2% nucleotide sequence identity, have a similar secondary structure, and both contain an ORF homologous to LAGLIDADG-type homing endonuclease with identical LAGLIDADG motifs (LAGLIEGDG and LAGFLDADG). By contrast, introns 1 and 2 in *P. angulospiculatus* share only 43.5% sequence identity in the aligned overlap regions and intron 1 does not contain any ORF.

Recently, group I introns highly similar to, and in the same position as intron 2 in *P. angulospiculatus* and its homolog in *Tetilla* sp. were reported in *cox1* of 20 scleractinian corals [24]. Phylogenetic analysis of amino-acid sequences derived from intronic LAGLIDADG ORFs in *P. angulospiculatus*, *Tetilla* sp., scleractinian corals, and several outgroup taxa grouped introns found in *Tetilla* sp. and *P. angulospiculatus* with 72% bootstrap support and placed them as a sister group to Scleractinian corals with 100% bootstrap support (Figure S2). The results of this analysis are consistent with the vertical evolution of this intron in cnidarians and sponges and suggest that its sporadic presence among sampled taxa is due to independent losses rather than the horizontal intron transfer proposed earlier [23]. This inference is reinforced by the observations that the genetic distance between LAGLIDADG ORFs in *P. angulospiculatus* and *Tetilla* sp. is similar to that between their host genes and that both ORFs contain a TGA codon at the same position (data not shown). The latter finding makes it highly unlikely that the two introns have been transferred in parallel from the nucleus, because TGA signifies a stop codon in cytoplasmic translation.

### rRNA genes

**Figure 6:**
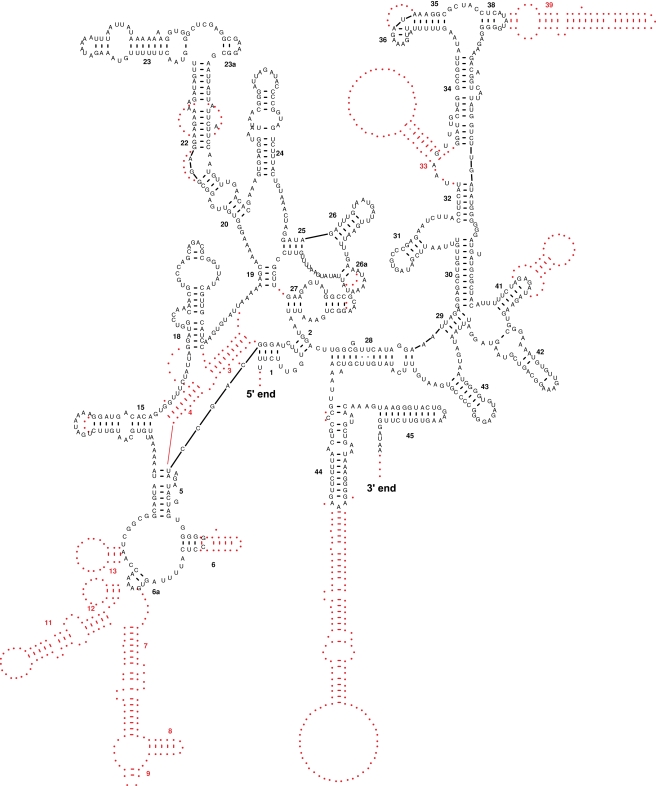
Inferred secondary structure of *Hippospongia lachne* mitochondrial small subunit RNA in comparison to that of *Oscarella carmela.* The helices are numbered in boldface as in Brimacombe *et al.* [52]. Structural regions present in *O. carmela* srRNA but absent in *H. lachne* srRNA are shown in red.

Genes for the small and large subunit ribosomal RNAs (*rns* and *rnl*) were located in close proximity of each other (separated by 1–3 tRNA genes) in most analyzed genomes, with the most common gene order being *+rns+trnG+trnV+rnl* (Figure 4). The two exceptions to this pattern were found in *Igernella notabilis,* where the two genes were separated by *atp9*, and *Oscarella carmela,* where *rnl* and *rns* were separated by multiple genes and had opposite transcriptional orientations. The size of *rns* ranged between 828 (*Hippospongia lachne*) and 1516 bp (*Ephydatia muelleri*), with the average size being 1224 bp. The size of *rnl* varied between 2166 (*Hippospongia lachne*) and 3487 bp (*Axinella corrugata*), with the average size being 2589 bp. The size differences in rRNA genes were due to two factors. First, some helices outside the core region of each rRNA were shortened or lost in several lineages, especially G1 (Figure 6). Second, unusual repetitive elements (see below) were inserted in rRNA genes in several distantly related species, in particular *Axinella corrugata*, *Ephydatia muelleri*, *Igernella notabilis*, and *Vaceletia* sp. (Figure S3).

### tRNA genes

Sampled demosponge mitochondrial genomes contained as few as 2 and as many as 27 tRNA genes. The variation in the number of tRNA genes was due to the loss of all but two mitochondrial tRNA genes (*trnM(cau)* and *trnW(uca)*) in G1, partial losses of tRNA genes in *Agelas schmidti* (at least one gene), *Amphimedon queenslandica* (at least 7 genes), and *Plakortis angulospiculatus* (at least 18 genes), the sporadic presence of *trnM(cau)e* among sampled species, and duplication of *trnT(ugu)* and *trnV(uac)* in *Oscarella carmela* mtDNA. Given that at least 24 species of tRNAs are needed for mitochondrial translation in demosponges [12], we expect that the loss of tRNA genes from mtDNA is compensated by the import of required tRNAs from the cytoplasm.

**Figure 7:**
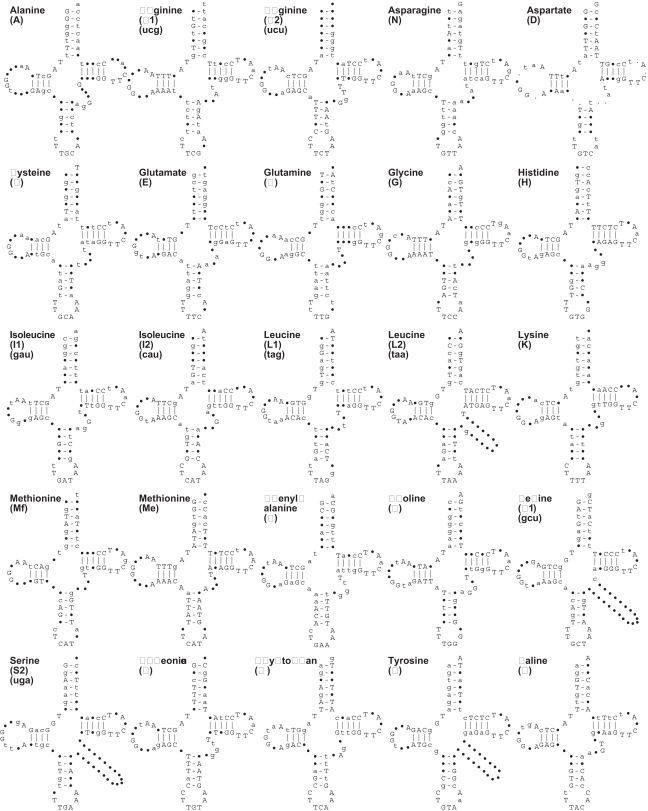
Secondary structures and consensus sequences of demosponge mitochondrial tRNAs. The secondary structure of each type of tRNAs was folded based on sequence and structure alignment. Nucleotides in uppercase letters indicate >90% sequence conservation, lowercase letters indicate >75% sequence conservation, and the dots represent <75% conservation.

In accord with our previous study [12], tRNA genes in all studied demosponge mtDNA were well conserved in terms of size, primary sequence and inferred secondary structure. All inferred mt-tRNA structures had well conserved D- and T-loops (7–11 and 7 nucleotides in length, respectively) with a potential to form the standard tertiary interactions G18-U55 and G19-C56. Variable or semi-invariable nucleotide positions, and secondary and tertiary interactions known for prokaryotic and nuclear tRNAs were also well conserved (Figure 7). At the same time, an unusual A11-T24 pair in 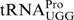 and an unusual G11-C24 pair in 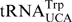 were present among all sampled demosponges. The first of them is characteristic for demosponges, glass sponges, and placozoans [12], [14], while the second–for all bilaterian animals [25]. The R11-Y24 pair is otherwise a distinctive feature of bacterial, archaeal, and organellar initiator 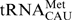 that is strongly counter-selected in elongator tRNAs [26].

Among individual tRNA genes, *trnW(uca)* had the most conserved primary structure (84.9% pairwise sequence identity on average) while *trnS(uga)* was the least conserved (66.7% identity on average). The inferred gene for elongator tRNA(M) (*trnM(cau)e*) that is present in 11 out of 22 analyzed genomes also displayed high sequence conservation (average pairwise identity 79.6%), an observation that suggests its intermittent occurrence among sampled genomes is due to multiple losses rather than *de novo* evolution through gene duplication and/or recruitment [e.g., 27]. Interestingly, the other gene for methionine tRNA (*trnM(cau)f*) is more conserved among the species where *trnM(cau)e* is present, than among species were it is absent (78.1% vs. 67.8% pairwise identity on average).

Our previous analysis discovered several cases of tRNA gene recruitment in *Axinella corrugata* [27]. The more expanded dataset of demosponge mitochondrial tRNA genes assembled for this study revealed several additional instances of tRNA gene recruitment in demosponge mtDNA (to be described elsewhere).

### Intergenic regions and repeats

The combined size of non-coding regions in the 22 demosponge mtDNAs analyzed in this study varied from 371 bp in *Geodia neptuni* to 6077 bp in *Axinella corrugata* or from 2 to 24% of the total genome size. In contrast to bilaterian animals, the distribution of non-coding nucleotides was more even in demosponge mtDNA, with the largest intergenic region usually containing <15%, and at most 39% (in *Iotrochota birotulata*), of all non-coding nucleotides. We found little conservation in the position of the largest intergenic regions among the sampled genomes, even for the species that share identical gene arrangements, such as *Chondrilla nucula* and *Halisarca dujardini*, *Geodia neptuni* and *Cinachyrella kuekenthali*, and *Hippospongia lachne* and *Vaceletia* sp. Furthermore, we detected little sequence conservation either among individual regions within each mtDNA or between identically located non-coding regions in different species, except for the presence of repetitive elements in some genomes, as described below.

Multiple repetitive elements were found in several analyzed genomes. Repeats larger than 100 bp were found only in *Vaceletia* sp., with the two biggest repetitive elements (229 bp) located in the intergenic regions that flank *nad2*, while 20–100 bp repeats were discovered in multiple species. The most abundant repeats were found in *Vaceletia* sp., *Igernella notabilis*, *Ephydatia muelleri*, and *Axinella corrugata,* where they have been located in most intergenic regions, as well as in ribosomal RNA genes and even some protein coding genes. The presence of repeated elements was very sporadic in respect to phylogeny, with repeats often present/absent in closely related species. Overall, repeats were very rare in sampled species from G0, G2 and G3, but more common in G1 and G4.

## Discussion

Our analysis of 22 complete mtDNA sequences representing all 14 orders of demosponges revealed both remarkable conservation and also an extensive diversity in mitochondrial genome organization within this group. Among the features shared among all sampled demosponge mitochondrial genomes are compact organization of the genetic material, similar gene content, well conserved structures of encoded tRNAs, a minimally modified genetic code for mitochondrial translation, and the absence of a single large “control” region characteristic of mtDNA in bilaterian animals. Genomic features that showed substantial variation include the number of tRNA genes, rRNA structures, the presence/absence of introns, and gene arrangements. In particular, two groups clearly stand out in our analysis with respect to their genome organization: G0 (order Homosclerophorida) and G1 (orders Dictyoceratida, Dendroceratida, and Verticillitida).

As reported previously, the mitochondrial genome of the homosclerophorid *Oscarella carmela* contains 44 genes–the largest complement of genes in animal mtDNA–including *tatC,* a gene for subunit C of the twin arginine translocase that has not been found in any other animal mtDNA, and genes for 27 tRNAs [14]. By contrast, the mtDNA sequence of the homosclerophorid *Plakortis angulospiculatus* determined for this study contains only 20 genes and lacks *tatC* as well as 19 of the 25 tRNA genes typical for demosponges. Other differences between these two genomes include distinct gene arrangements (only 4 shared gene boundaries) and the presence of two group I introns in *P. angulospiculatus cox1*. Furthermore, the estimated genetic distances between these two species are greater than those between many orders of demosponges, indicating an ancient radiation and the presence of extensive genetic diversity within this group.

Mitochondrial genomes of the three species within the G1 group are also unusual. These genomes lack all but two tRNA genes (for methionine and tryptophan tRNAs)–a feature previously associated with cnidarian mtDNA [28]. Furthermore, this is the only group of demosponges where a significant acceleration in the rates of mitochondrial sequence evolution has been detected. There appears to be no causal connection between these two observations, as the loss of all but two tRNA genes is shared by all three species in the group, while the accelerated sequence evolution is much more pronounced in Dictyoceratida and Verticillitida. The retention of *trnW(uca)* and *trnM(cau)* as the only tRNA genes in the genome supports our previous inference [29] that these genes are difficult to replace because of the unique role of their products in mitochondrial translation: 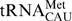 is used for the initiation of mitochondrial translation with formylmethionine [30] while 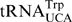 must translate the TGA in addition to the TGG codons as tryptophan. The presence of such constraints can cause a parallel genomic evolution in independent lineages.

An unusual mitochondrial genome has been previously reported for the haplosclerid demosponge *Amphimedon queenslandica* [15]. This genome lacks *atp9* and at least seven tRNA genes, contains deletions in several protein coding genes, and displays accelerated rates of sequence evolution in both protein and RNA genes. Our analysis of three additional species from the same order, *Amphimedon compressa*, *Callyspongia plicifera,* and *Xestospongia muta*, found no similar features in the latter taxa. These results most likely indicate that *A. queenslandica* mitochondrial genome has undergone an unusual evolution and is a poor representative of the G3 group, although incorporation of nuclear sequences, such as nuclear Numts [31], in the mtDNA assembly cannot be ruled out. Given that *A. queenslandica* has become a model system for the study of demosponge biology, the evolution of its unusual mtDNA should be investigated in more details.

Another interesting result that came out of this study is the discovery of two group I introns in *cox1* of *P. angulospiculatus*. Several lines of evidence, including phylogenetic analysis, the identical location in *cox1*, a similar extent of genetic divergence to their host genes, and the presence of TGA codons at the same position, support the vertical evolution of one of these introns from the common ancestor shared not only with *Tetilla* sp. (order Spirophorida), but also with scleractinian corals. This in turn suggests that the absence of this intron in most demosponge lineages is due to massive parallel loss. While examples of such losses are well known in nuclear genomes [32]–[37], an interesting question posed by this result is why mitochondrial introns are retained so scarcely in demosponges but so commonly in cnidarians?

Finally, this study is interesting in what we did not find–any structures and/or sequences potentially involved in the maintenance and expression of mtDNA. Obviously, replication and transcription initiation/termination signals do exist in these genomes, but they were not detected by our comparative genomic analysis. Further data collection and experimental work will be essential to elucidate the mechanisms of these processes in demosponge mitochondria.

## Methods

### Genome sequencing and phylogenetic analysis

Taxon sampling, DNA extraction, PCR amplification, and sequencing were described in our previous article [11]. Phylogenetic analysis of demosponge relationships was conducted with the PhyloBayes program [38] as described previously [11], except that mitochondrial sequences from several taxa (mostly cnidarians) have been added: *Agaricia humilis* NC_008160, *Anacropora matthai* NC_006898, *Aphrocallistes vastus* EU000309, *Branchiostoma floridae* NC_000834, *Capsaspora owczarzaki*, *Colpophyllia natans* NC_008162, *Discosoma* sp. CASIZ 168915 NC_008071, *Hydra oligactis* EU237491, *Montipora cactus* NC_006902, *Mussa angulosa* NC_008163, Placozoan sp. BZ2423 NC_008834, Placozoan sp. BZ49 NC_008833, *Pocillopora damicornis* NC_009797, *Porites porites* NC_008166, *Pseudopterogorgia bipinnata* NC_008157, *Rhodactis* sp. CASIZ 171755 NC_008158, *Seriatopora caliendrum* NC_010245, *Siderastrea radians* NC_008167.

### Annotation and analysis of coding sequences

We used flip (http://megasun.bch.umontreal.ca/ogmp/ogmpid.html) to predict ORFs in assembled sequences; similarity searches in local databases and in GenBank using FASTA [39] and NCBI BLAST network service [40], respectively, to identify them. Protein-coding genes were aligned with their homologues from other species and their 5′ and 3′ ends inspected for alternative start and stop codons. Inferred amino acid sequences of encoded proteins were aligned with ProbCons [41] using default parameters. Genetic distances between demosponges and four species of cnidarians (*Briareum asbestinum, Metridium senile, Montastraea annularis* and *Ricordea florida*) were calculated with the TREE-PUZZLE program [42], using the mtREV matrix, estimated frequencies of amino acids and 8 gamma rate categories. Effective numbers of codons [43] were calculated with the chips program within the EMBOSS package [44].

### Annotation and analysis of RNA genes

Genes for small and large subunit ribosomal RNAs (*rns* and *rnl*, respectively) were identified based on their similarity to homologous genes in other species, and their 5′ and 3′ ends were predicted based on sequence and secondary structure conservation. The secondary structures of selected rRNA genes were manually folded by analogy to published rRNA structures, and drawn with the RnaViz 2 program [45].

Transfer RNA genes were identified by the tRNAscan-SE program [46] and aligned manually in MacGDE 2.3 [47] using their secondary structure as a guide. This alignment was used to calculate sequence conservation at each position and average pairwise identity values for individual tRNAs. For the latter calculation we excluded all tRNAs from *Plakortis angulospiculatus, Amphimedon queenslandica* and all species in G1, which encode incomplete sets of tRNAs in their mtDNA.

### Intronic sequences

We used intron prediction programs RNAweasel [48] and Rfam [49] to search for introns in coding sequences. The exact positions of two introns found in *cox1* of *Plakortis angulospiculatus* were adjusted based on *cox1* alignments with homologous sequences from other demosponges. The inferred amino acid sequence of the large ORF found in one of the *P. angulospiculatus* introns was aligned with the sequences of LAGLIDADG ORFs analyzed by Rot *et al.* [23] and Fukami *et al.* [24] and used for a phylogenetic analysis. We selected the best model for these ORFs with the ProtTest program [50] and performed a maximum likelihood search and bootstrap analysis in TREEFINDER [51], using the WAG model of sequence evolution, estimated amino acid frequencies and 4 gamma categories.

### Intergenic regions and repeated sequences

Intergenic regions were extracted from each genome with the PEPPER program (http://megasun.bch.umontreal.ca/ogmp/ogmpid.html) and searched for similarity using FASTA. In addition, we searched for interspersed identical repeats in individual genomes using FINDREP (http://megasun.bch.umontreal.ca/ogmp/ogmpid.html) with minimum repeat subsequence lengths of 20 bp and 100 bp respectively.

## Supporting Information

Figure S1 (0.29 MB EPS)

Figure S2 (0.24 MB EPS)

Figure S3 (0.69 MB EPS)

Table S1 (0.04 MB XLS)

Table S2 (0.04 MB XLS)
